# Mothers’ Access to Social and Health Care Systems Support during Their Infants’ First Year during the COVID-19 Pandemic: A Qualitative Feminist Poststructural Study

**DOI:** 10.3390/nursrep13010038

**Published:** 2023-03-07

**Authors:** Britney Benoit, Megan Aston, Sheri Price, Damilola Iduye, S Meaghan Sim, Rachel Ollivier, Phillip Joy, Neda Akbari Nassaji

**Affiliations:** 1 Rankin School of Nursing, St. Francis Xavier University, Antigonish, NS B2G 2W5, Canada; 2 School of Nursing, Dalhousie University, Halifax, NS B3H 4R2, Canada; 3 Research, Innovation & Discovery, Nova Scotia Health, Halifax, NS B3S 1B8, Canada; 4 Department of Applied Human Nutrition, Mount Saint Vincent University, Halifax, NS B3M 2J6, Canada; 5 School of Nursing, Abadan University of Medical Sciences, Abadan 631, Iran

**Keywords:** postpartum, mothers, infants, COVID-19, health system, social support, parenting, qualitative, feminist poststructuralism, discourse analysis

## Abstract

Social support and health services are crucial for mothers and families during their infants’ first year. The aim of this study was to explore the effect of self-isolation imposed by the COVID-19 pandemic on mothers’ access to social and health care systems support during their infants’ first year. We utilized a qualitative design using feminist poststructuralism and discourse analysis. Self-identifying mothers (*n* = 68) of infants aged 0 to 12 months during the COVID-19 pandemic in Nova Scotia, Canada completed an online qualitative survey. We identified three themes: (1) COVID-19 and the Social Construction of Isolation, (2) Feeling Forgotten and Dumped: Perpetuating the Invisibility of Mothering, and (3) Navigating and Negotiating Conflicting Information. Participants emphasized a need for support and the associated lack of support resulting from mandatory isolation during the COVID-19 pandemic. They did not see remote communication as equivalent to in-person connection. Participants described the need to navigate alone without adequate access to in-person postpartum and infant services. Participants identified conflicting information related to COVID-19 as a challenge. Social interactions and interactions with health care providers are crucial to the health and experiences of mothers and their infants during the first year after birth and must be sustained during times of isolation.

## 1. Introduction

Maternal, newborn, and child health are global health priorities. Postpartum care and health services need to be responsive and accessible to mothers and their infants. International policy recommendations and initiatives call for early and unified postpartum intervention to ensure the short and long-term physical, emotional, and social well-being of mothers, families, and children [1,2]. In particular, timely access to social support and health care services has been identified as a priority for new parents to improve outcomes [1]. Social interactions have been identified as an essential form of support for new parents [3,4,5]. However, the mandatory isolation imposed by the COVID-19 pandemic has led to unprecedented changes in the support and social interactions available to families and has resulted in rapid shifts in health systems and service approaches, availability, and accessibility.

The postpartum period is a time of great transition and stress for mothers and families. The potential for negative health outcomes exists if the needed supports and resources are not available [1,2]. In Canada, policy recommendations and government initiatives encourage programs and services for mothers and families, particularly those who face challenging life circumstances, to promote physical, emotional, and social well-being [6,7,8,9,10,11]. As described in qualitative studies conducted in the United States of America [12] and Belgium [13], and a qualitative synthesis including studies conducted globally [14], various avenues to access support and information are available in the postpartum period. These include public health services, home visitation programs, physician and primary care provider offices, midwives, family resource centers, grass roots drop-in centers, private online forums, and informal conversations with other parents.

In studies on health outcomes of mothers in the postpartum period, researchers have focused on measures such as breastfeeding rates, hospital admissions, and physical health indicators of mothers and infants (e.g., rates of preterm birth, maternal-infant morbidity and mortality, and cesarean birth) [15]. While these outcomes are significant, mothers also reported that parental confidence, parenting ability, and stress were important, but these factors are less often a focus in research [16]. Researchers reported that mothers want and need validation and reassurance about their parenting practices in person from other mothers and health care providers whom they deem as trustworthy and non-judgemental, [16,17]. In-person interaction was perceived to better enable interpersonal connection and social support when compared to virtual options [17]. Social support encompasses tangible and psychological resources to support self-efficacy, self-esteem, and subsequent coping in response to challenges [18]. Aston et al. [16,19] and Price et al. [17] showed that social support for new parents that includes empathy and sharing of ideas and information with other parents and health care professionals (online and in person) helps parents build confidence. Other researchers found that social support was essential to improve outcomes related to breastfeeding and maternal mental health [3,4,5,20,21,22,23]. However, the complex relational, social, and experiential aspects of the postpartum period are often deemphasized, devalued, and unprioritized within the health care system [24,25].

Given the integral role of social support, resources, and health services for mothers and families during their infants’ first year, social isolation can put families and infants at risk for poor physical and mental health outcomes [26,27]. The Canadian social and health system’s response to the COVID-19 pandemic led to public health orders, including unprecedented lockdown and isolation, to minimize infectious disease spread. However, there is no evidence to guide the implementation of health services and social supports that meet families with infants’ needs in this novel and rapidly changing context. Significant alterations in delivery and access to a multitude of face-to-face pregnancy and postpartum supports have occurred [26,28,29,30]. The Canadian response to COVID-19 provides an opportunity to examine how families experience caring for an infant in isolation. Understanding these experiences is needed to inform evidence-based support and resources that are responsive to family needs and promote mental and physical health and well-being. The purpose of our study was to explore the effect of self-isolation in response to the COVID-19 pandemic on mothers’ access to social and health care systems support during their infants’ first year. We explored how mothers experienced their infants’ first year; how the COVID-19 pandemic affected access to support, including health care services; and how mothers constructed their experiences personally, socially, and institutionally.

## 2. Materials and Methods

### 2.1. Design

We used a qualitative design guided by feminist poststructuralism as described by Weedon [31]. This approach was selected as it provides a way to examine how participants’ experiences were personally, socially, and institutionally constructed through different subjective positions such as gender, race, ethnicity, sexual orientation, class, socio-economic status, culture, and abilities [31,32,33,34]. Feminist poststructuralist methodology prompts us to examine how different beliefs, values, and practices are constructed through relations of power across individuals, institutions, and social contexts [31,32,33,34,35]. Discourse analysis [31,36] was used to examine and deconstruct meaning in the language of participants’ personal experiences. Members of our research team have previously used feminist poststructuralism and discourse analysis to examine social justice issues in the health care system through the exploration of subjective positions and their influence on beliefs, values, practices, and relations of power between health care providers and patients in maternal and infant health [16,17,19,24,37]. Feminist poststructuralism thus provides a relevant lens through which to examine mothers’ experiences of their infants’ first year during the mandatory isolation imposed due to COVID-19. The Consolidated Criteria for Reporting Qualitative Research (COREQ) was followed.

### 2.2. Setting

In Nova Scotia, Canada, the study setting, a state of emergency was declared because of COVID-19 beginning on 22 March 2020. Individuals were instructed to isolate in their homes and to cease all non-essential activities outside of the home. Nova Scotia also required that all individuals who traveled from outside of the province complete a mandatory self-isolation for 14 days upon entering the province, which limited the ability to travel inter-provincially. Health care services were limited to essential and emergency services. Restrictions were put in place to limit visitors to in-patient settings, and virtual care was implemented in many contexts, such as primary care. Public health programming shifted to COVID-19 response and a hold was placed on postpartum follow-up.

### 2.3. Participants

Self-identified mothers of an infant aged 0 to 12 months during the initial time of the COVID-19 pandemic (March–June 2020) and were able to read and write in English were eligible to participate in the study. Participants with diverse childbirth experiences were considered eligible, including adoptive mothers, mothers who experienced cesarean or vaginal birth, mothers of full-term or preterm infants, and mothers of hospitalized neonates.

### 2.4. Data Collection

We obtained ethics approval from the Research Ethics Board of IWK Health (May 2020). We recruited a convenience sample through social media platforms Twitter, Facebook, and Instagram. We shared social media posts that specifically targeted mothers in Nova Scotia who gave birth during the early COVID-19 pandemic on our research team’s website (www.mumsns.ca (accessed on 26 February 2023)) and social media platforms (i.e., Twitter, Facebook, Instagram). We also shared the recruitment notice with provincial family resource centers, parenting groups, and health care organizations. We collected data using an online qualitative survey directly hosted on our research team’s secure website. Potential participants reviewed an online letter of introduction and completed the consent form by checking a consent box before proceeding to the survey. We maintained participant confidentiality by removing all identifying information from survey responses and by using password-protected files. We only included non-identifying demographic data (such as age, ethnicity, sexuality, number of children, and household income) in the online questionnaire. Participants were not able to withdraw study data once they submitted their questionnaires. Aligned with our methodology [31] and previous research conducted by our research team [16,17], the survey consisted of three open-ended questions: (1) Tell us about your experience at home with your new baby and how the situation created by the COVID-19 pandemic affected you and your family, (2) tell us about your experience of support (from friends, family, health care professionals, etc.) during the COVID-19 pandemic and (3) tell us about your experience of searching for and receiving information about caring for yourself, your baby, and your family during the COVID-19 pandemic.

### 2.5. Data Analysis

Team members who completed the analysis were from diverse socially constructed positions including race, gender, and sexual orientation. This supported a diversity of perspectives when interpreting the data. We specifically followed the data analysis steps recommended by Aston [32] which integrates foundational literature on feminist poststructuralism and discourse analysis [31,33,34,36] to guide the feminist poststructural analysis of qualitative data. These data analysis steps include: (1) identify major issues, (2) apply beliefs, values, and practices, (3) write about social and institutional discourses (i.e., the beliefs, values, and practices of the social and institutional contexts), (4) respond to relations of power, and (5) add participant subjectivity and agency. Each of the eight team members read the participant transcripts individually, focusing on what each participant had written to describe the individual meaning of their experience. We first started by identifying each participants’ beliefs and values as they related to their experience, followed by what they did or ‘practiced’ in response to that experience. For example, if a participant described anger at the lack of physical support for breastfeeding due to COVID-19 related health systems changes, we closely reviewed the language of the participant to understand their personal beliefs and values related to physical breastfeeding support (such as physical breastfeeding support is important and should be available). We then went on to identify how the beliefs, values, and practices of social and institutional context informed the participant’s experience using the words and meanings described by the participant. For example, participant responses highlighted that there was a social/institutional belief that it was not important for postpartum mothers to have access to breastfeeding support, resulting is these services being stopped due to COVID-19. We then went on to identify participants’ subjective experiences, how they responded to the relations of power in the social/institutional context, and if they exercised agency to practice their beliefs and values related to accessing information and support during the postpartum period (i.e., did participants challenge the social/institutional belief that support for breastfeeding is unimportant? How did they do so?). The individual personal experiences of participants were the foundation of our analysis which then led to examining similarities and differences in experiences across all 68 participants. Discourse analysis requires the inclusion of a variety of concepts that cannot be linearly organized in software data. Therefore, we used an iterative back and forth way of discussing meaning until there was consensus [32]. We iteratively discussed the identified participant beliefs, values, and practices; the impact of social and institutional context; relations of power; and participant subjectivity and agency. This discussion allowed us to come to a consensus on emerging themes grounded in feminist poststructuralism that best represented participant experiences from their responses.

This paper presents a secondary analysis of data from a previously published report [37]. The analysis presented in this paper is unique from our previous reports in that its focus is specifically on the personal, social, and institutional construction of mothers’ experiences of health systems support and social support.

### 2.6. Trustworthiness

We maintained trustworthiness through accurate documentation of participant responses and detailed notes [38,39]. We attained credibility, dependability, and confirmability by investigator triangulation through regular team meetings to discuss and confirm the ongoing analysis. Team members used reflexive notes to identify personal observations, feelings, and biases [38]. As part of the feminist poststructural analysis, we discussed our own socially constructed positions and their influence on the analysis to enhance credibility and authenticity [32]. Dependability and confirmability were established through an audit trail that recorded analysis decisions and rationale.

## 3. Results

Participant demographic information is summarized in Table 1. All participants (*n* = 68) were from Nova Scotia, Canada. All participants identified as mothers and were predominantly White heterosexual women who lived with their partners. Approximately half of the participants were first-time mothers.

We identified three distinct themes that captured participants’ experiences of isolation during the COVID-19 pandemic and their health care and social support needs: (1) COVID-19 and the Social Construction of Isolation, (2) Feeling Forgotten and Dumped: Perpetuating the Invisibility of Mothering, and (3) Navigating and Negotiating Conflicting Information.

### 3.1. COVID-19 and the Social Construction of Isolation

As captured under the first theme, COVID-19 and the Social Construction of Isolation, participants described how isolation negatively affected their experiences in extreme ways due to public health orders to stay home and not visit with members of other households. Most participants spoke about how their needs for social-emotional and practical support to care for an infant and themselves were unmet because they were isolated at home. Connecting with the community was identified as very important in the postpartum period. Many mothers experienced tension between the expressed need for community and the restrictions in place limiting that community. This statement exemplifies the extreme effect that public health orders had on many participants in this study:

*There is no support. While text messages and phone calls help, it’s not the same. It took me a while to be less angry at the adage that it takes a village to raise a family; that village is illegal.*

(Participant 4)

Raising children and families together with others outside one’s immediate family was important to this participant, as well as others in our study. Being denied access to their broader communities produced feelings of anger. Participant 4′s statement highlights the emotive aspect of isolation that can be devastating for families. Our analysis revealed that the meaning of community was constructed through a discourse that valued and expected families to support and ‘raise’ each other. Participant 4 demonstrated how she used her agency to question how the community was being handled by public health. If we see the relation of power as constructed between the public health mandate for isolation and the desires of parents for the community, then we can see participant 4 challenging the systemic experience of isolation. Feeling isolated and alone in such an extreme way with a new infant was stressful and highlighted the need to be connected to the larger community to feel supported:

*It has been hard to balance my day to keep him busy and thriving. I feel as if I am a bad mum for allowing him independent play time, which often involves his books, we do not watch or use technology. He has become more demanding of my time, and is able to open up all the drawers/cupboards. Meeting with other mums and dads means that I can learn new ways to help him grow and develop.*

(Participant 13)

Overwhelmingly, participants stated that isolation was detrimental to their health and the health of their infants. To break the isolation, most participants used virtual technology to connect socially and seek information and support from family during the pandemic. However, remote communication using phone or video communication programs was not seen as equivalent to in-person connection, physical contact, and support. While several participants used the phone to call family for advice, the need for practical help (e.g., groceries) and support in the day-to-day tasks of caring for a new infant could not be met through virtual contact. Several participants spoke about the challenges associated with virtual contact, such as issues with scheduling with family members:

*I have very little support, sometimes my family FaceTimes, but my husband is at work all day and both of our families live in separate areas. […] I can talk to people on the phone but everyone I know is still working […] Meaning that I feel even more isolated as I have not had a face-to-face conversation with anyone aside from my pharmacist since the state of emergency began.*

(Participant 13)

Phone and FaceTime were the primary tools participants used to connect with family and friends. Many expressed that while this was helpful, it also reminded them of what they were missing. They believed that it was imperative that they connect with people in-person. Mother-baby yoga and swim groups, walks in the park, play dates, library visits and reading sessions, and music classes were all viewed as engagement opportunities that were essential to social and emotional health. One participant described how her mental health was suffering because of her lack of connections with other mothers:

*I feel the biggest difficulty has been finding social support for my emotional health. I had wanted to find a group of local moms pre-pandemic and had found yoga, stroller fit, pelvic floor physio, music, and swim groups that I wanted to try out for bonding with my daughter and expanding my support circle. That has been the most difficult hole to fill by far and I feel my mental health is suffering because of missing connection with other first-time moms.*

(Participant 8)

Many participants spoke about the pressure to “be everything” for their new baby without the broader community of social support. This demonstrates the social discourse that has been constructed that mothers should be everything for their babies and know how to ‘mother’ or ‘parent’ naturally [40]. This creates pressure and compounds the experience of isolation. The assumption that mothers are to be everything for their babies counteracts and minimizes that belief that mothers need to connect with and be supported by the community (through meeting other parents or receiving support from nurses and other health care providers). Participant 8 challenges the notion of ‘being everything’ when she articulates that she believed she could bond with her daughter through meeting with other people. This challenges the discourse that bonding is an individually constructed experience and a private matter between mother and child. Another participant shared an example of the pressure isolation placed on parents:

*The pandemic has limited our social networks (i.e., friends and other moms/parents) and our in-person support network (i.e., family), and increased the pressure on us as new parents to provide care, support, interaction, socialization, and entertainment for our child and one another.*

(Participant 16)

We can see that the pandemic exacerbated the normalized discourse of postpartum isolation and the expectation that mothers and parents be alone with their babies. Participants tried different ways to deal with isolation such as trying to connect with family, friends, and health care providers by telephone and various online platforms. While this new way of connecting was helpful in some ways, it was not ideal for most participants.

### 3.2. Feeling Forgotten and Dumped: Perpetuating the Invisibility of Mothering

Under the second theme, Feeling Forgotten and Dumped: Perpetuating the Invisibility of Mothering, participants emphasized the effects of the COVID-19 health system response on the postpartum care of mothers and infants. While some believed that their health care needs were met by the health system, most emphasized how changes in service delivery due to the pandemic response did not meet their social, emotional, or physical needs. Participants described essential postpartum and newborn services being eliminated completely or reduced to virtual care because of public health orders to minimize physical contact. This left most participants feeling they had to navigate alone without routine care and were left to decide what was physically and emotionally normal for them and their infant. Many participants specifically spoke about feeling forgotten by the health system:

*I also feel as if moms are being treated as second class citizens as they no longer offer 6 weeks postpartum checkups meaning that we are left to decide if things seem normal or not after giving birth.*

(Participant 27)

*My 6-week postpartum appointment became over the phone. I had a second-degree tear and my stitches had opened once returning home. Since I had a phone appointment, I had nobody check and tell me if I was healing properly.*

(Participant 62)

Some participants felt that this shift to virtual care met their information and support needs and viewed virtual care as a way of reducing their risk of COVID-19 exposure. However, most emphasized that virtual care was not meeting their needs and described how the gaps created by virtual care were compromising both their health and the health of their infants. The relationship between mothers and their health care providers was significantly altered due to mandated isolation. Mothers still expected their health care providers to be experts who would be able to let them know if things were normal and if they were healing properly. However, connecting virtually did not support this expected relationship. The gap in care was particularly evident in relation to breastfeeding support:

*Our daughter had a tongue tie and we were initially connected to public health, lactation consultants, and the collaborative breastfeeding clinic. We had one meeting with public health before they reassigned our nurse to the COVID team and we were dumped. The breastfeeding clinic cut her tongue tie but did a phone follow up instead of in person due to COVID and dumped us even though we were still having feeding issues. The lactation consultants at the [hospital] also closed our file early due to COVID […] I’ve felt very let down by the health care system.*

(Participant 8)

Feeling ‘like a second-class citizen’, ‘let down’, or ‘dumped’ clearly demonstrates that participants felt forgotten and that negotiating relations of power associated with the shift to virtual care was extremely difficult for them. Participants challenged virtual care by expressing their belief that it was unacceptable to be denied help from their health care providers and to be expected to deal with postpartum issues on their own.

### 3.3. Navigating and Negotiating Conflicting Information

As described under the third theme, Navigating and Negotiating Conflicting Information, there was a lack of information and conflicting recommendations and advice regarding COVID-19 and its potential effects on infant health, maternal health, breastfeeding, and the safety of childcare. Participants identified continual staffing changes associated with the health system response to COVID-19 as contributing to conflicting information and confusion about where to go for information. It was clear that participants in our study had a very difficult and often stressful time searching for reliable information and advice. They were in a position of questioning the health care system and trying to negotiate the constant changes and shifting power due to COVID-19. While some participants could not confidently choose between conflicting advice, others spoke about how they demonstrated agency to critically evaluate information from health care providers and other sources. Lack of access to health care providers, family, friends, and other parents to discuss and critique different information made conflicting recommendations particularly frustrating and problematic:

*It was a hassle trying to figure out who would do my daughter’s 2 months needles [injections] as the public health nurse told me she did not need them, and that she would be protected by herd immunity. Knowing this is not appropriate information to give people, we found someone to immunize her.*

(Participant 62)

This participant did not trust the health professional advice they received regarding immunizations, so they searched for and appraised information on their own. Most participants attempted to access information and support from health care providers, however, many participants also sought and accessed information through non-professional, online sources. Some participants perceived Google as a valuable resource, whereas others perceived knowledge accessed in this way as variable, stressful, extreme, or not relevant. Most mothers found it was an ongoing challenge to search for information and decide what information they would trust:

*I think the biggest struggle at the moment is finding information related to the risk of sending our son to daycare. To me, it seems preposterous that it is too dangerous for me to go into the office to work but it will be safe to send my 12-month-old son to daycare which is known for being high [risk] for sickness during non-COVID-19 times.*

(Participant 1)

This statement highlights the struggle that many participants experienced between their own personal knowing and available knowledge related to the health of their baby. They sought validation and reassurance regarding systems-level decisions being made that impacted their own and their infant’s life.

## 4. Discussion

Our findings provide insights into the postpartum experiences of Canadian mothers during the COVID-19 pandemic that can inform evidence-based support and services. Participants emphasized the importance of community and social support during the postpartum period and described that in-person connection was necessary but also unavailable due to the pandemic. This finding is consistent with other research where participants identified that a lack of in-person support in the postpartum period from family, friends, and health care providers challenged their physical, social, and emotional well-being [13,41]. Our analysis shows that the meaning of community was constructed by most participants through values and beliefs that families ‘raise’ each other as part of a village of support. Participants also identified the competing social and institutional discourse that mothers should be able to cope ‘naturally’ with a baby on their own. Social isolation may be seen as a ‘normal’ and a good thing for mothers and infants to bond together [40]. However, previous researchers have shown that the postpartum period is an isolating time for new mothers [13,41,42] and mothers do not feel psychologically and practically supported in the postpartum period [13]. Participants in our study echoed these experiences, highlighting how the social restrictions and lack of health care provider availability that resulted from the COVID-19 pandemic increased their experiences of isolation, loneliness, and lack of support. Feeling like they had not been given priority treatment and using terms like “second-class citizen”, participant responses highlight how to COVID-19 pandemic exacerbated the invisibility of postpartum care and mothering.

Support for mothers and parents has been constructed through different discourses. A health discourse of support primarily focuses on physical and psychological support constructed through practices of meeting with health care professionals [43] and is based on a relationship of health care provider as the expert and the mother as the client or consumer. A community discourse of support focuses more on psychological and emotional support through practices of meeting with other mothers/parents, family, and friends [16,17,19]. Community discourses of support are often constructed as less important than health care provider support that is focused on physical outcomes. Our participants believed that social, emotional, and physical support from family and friends, as well as health care professionals, was crucial to their own and their family’s wellbeing, demonstrating the importance of both discourses. In a recent meta-synthesis of 36 qualitative studies across 15 countries, Finlayson et al. [14] identified practical and emotional support from partners, families, and elders (*n* = 18 studies); information, advice, and support from health care providers (*n* = 16 studies); and information, support, and reassurance from other mothers (*n* = 15 studies) as highly important to mothers in the postnatal period. Previous work has shown that mothers particularly value and trust the advice and support they gain from in-person interactions with other mothers who have infants of a similar age [16,17]. Participants in our current study described how the COVID-19 related restrictions diminished social networks and created situations of extreme isolation that prevented them from engaging with other mothers and infants. This brings to light how a mothering discourse perpetuates the undervalued complexities of raising an infant.

Participants described in-person interactions with care providers as essential. Participants acknowledged that while virtual care was helpful at times, it did not meet all physical, emotional, and informational care needs. Furthermore, inconsistent information related to infant care and health in the context of the COVID-19 pandemic from health care providers and online sources was a source of stress. The construction of knowledge related to the postpartum period and mothering can take on many forms through different discourses. Medical, health, community, or grassroots are some of the discourses that continue to be perpetuated. There is still an expectation that postpartum information can and should be obtained from health care ‘experts’ [43,44]. Many participants in our study identified cessation of services or lack of access to in-person appointments with health care providers as a “let down” and felt that they were forgotten to navigate caring for an infant on their own. This highlights the value mothers place on consulting with ‘experts’ to access consistent information to meet their physical needs in the postpartum period, primarily related to their physical care, infant development, immunizations, and breastfeeding and infant nutrition [13,14]. However, the participants in our study also challenged the ‘health care provider as expert’ discourse when presented with conflicting information and exercised their agency to access the information they needed to feel confident in their parenting decisions. While this finding is consistent with our prior research [16,17,44], challenges with the negotiation of knowledge were exacerbated by having a new layer of information related to COVID-19 and its effect on maternal and infant health that was either not available or presented conflicting points of view. Participants in our study described having to rely even more on their own personal knowing and critique of information.

### Limitations

While we broadly recruited participants through social media platforms, our sample was largely homogenous and made up of White, heterosexual, female-identifying mothers with high employment incomes from Nova Scotia, Canada. Our findings may not reflect the experiences of diverse mothers, including those identifying as members of historically underrepresented groups (e.g., Black, Indigenous, racialized, neurodivergent, 2SLGBTQ+, persons with physical disabilities), families living with lower employment incomes, fathers, those who did not have access to social media or online technology to complete the survey, or those living outside of Nova Scotia, Canada. Purposive sampling of diverse participants is needed to capture those experiences. We did not characterize the clinical details of the sample (e.g., mode of birth, gestational age at birth, neonatal critical care experience). Therefore, it is not possible to draw conclusions regarding the influence of these factors on the experiences of the participants.

## 5. Conclusions

Our research highlights the personal experiences of mothers following childbirth surrounding the social and health system’s response to the COVID-19 pandemic. Social and health care provider interactions were crucial to the experiences of mothers and their infants. Governments and health systems must recognize the physical and psychological needs of mothers with infants during responses to public health crises. Opportunity for peer and health care provider interaction and support must be sustained during times of isolation to support health and well-being. Continued research to develop care approaches to best meet the social and health care needs of mothers raising their infants in isolation is necessary to support positive outcomes.

## Figures and Tables

**Table 1:** Characteristics of study participants (*n* = 68).

Characteristics	Frequency (Percent)	Mean/SD or Min-Max ^1^
Age (years)		31.6 ± 4.9
<25	5 (7)	
25–35	44 (65)	
>35	19 (27.9)	
Ethnicity		
White Black	63 (97) 0 (0)	
Mixed	2 (3)	
Sexuality		
Heterosexual	56 (93)	
2SLGBTQ (two-spirit, lesbian, gay, bisexual, trans, queer)	4 (7)	
Living status		
living with partner	63 (93)	
Single parents	5 (7)	
Place of residence		
City or town	53 (78)	
Village or small community	15 (22)	
Birth or adoption of baby		
Birth	68 (100%)	
Adoption	0	
Infants’ age (months)		6.1 ± 3.1
<6	27 (40)	
≥6	41 (60)	
Multiple child family		
Yes	33 (49)	
No	35 (51)	
Other children’s age (years)		5.6 ± 3.6
Number of other children in each family		[1–3]
Annual household income (CAD)		95,466 ± 54,983
<50,000	11 (18)	
50,000–100,000	27 (43)	
>100,000	24 (39)	

Note. Categories may not sum up to the total number due to missing data. ^1^ SD (Standard Deviation); Min-Max (Minimum-Maximum).

## Data Availability

Data are unavailable due to ethical restrictions.
